# The College Very Much Alive

**Published:** 1921-02-05

**Authors:** 


### The College Very Much Alive.

No recorded meeting of the College of Nursing has given greater confidence that the new professional spirit is healthily alive and growing, than that held at Guy's Hospital on January 25. The London Centre is wide awake, and has done admirable service in focussing attention on the need for better representation on the Council of interests and areas, and in pointing out the way in which this defect may be remedied. A strong consensus of opinion has made itself felt in the direction of strengthening the nomination system, and we are persuaded that, with the reform of nomination and the adoption by various centres of particular candidates, an entirely new interest will be imported into the business of election. The complete breakdown of the old *laissez-faire* system is evidenced by the fact, brought out by Miss Biggar, that, though 4,000 more voting-papers were sent out in 1920 than in 1919, 1,000 fewer were returned. On the vigour with which this apathy can be countered the future, not of the College alone, but of the General Nursing Council, can be plainly seen to depend.

The London Centre is doing very well as regards finance, and this may be called the sign and seal of success in these hard times. They have already begun to invest a surplus, and have War Loan to the amount of £2,650. An increase of 315 members in nine months is a good augury for future prosperity. The same note of financial stability echoed in Miss Rundle's remarks. It is matter for most sincere congratulation that 5,000 of the founder members have already become annual subscribers of from one guinea to half-a-crown. If another 5,000 become annual subscribers, as is anticipated, before the end of the financial year, March 31, the College will be in possession of a fixed annual income of between £2,000 and £3,000, and will be set free to accomplish, without fear of debt, many essential tasks for the profession at large. To be a subscribing member is to participate in a great national work—nothing less than the building up into a corporate whole of the finest profession open to all women.

